# Unlocking Phenolic Potential: Determining the Optimal Grain Development Stage in Hull-Less Barley Genotypes with Varying Grain Color

**DOI:** 10.3390/foods13121841

**Published:** 2024-06-12

**Authors:** Iván Friero, Alba Macià, Maria-Paz Romero, Ignacio Romagosa, Mariona Martínez-Subirà, Marian Moralejo

**Affiliations:** AGROTECNIO-CERCA Center, Universidad de Lleida, Av. Alcalde Rovira Roure, 191, 25198 Lleida, Spain; ivan.friero@udl.cat (I.F.); alba.macia@udl.cat (A.M.); mariapaz.romero@udl.cat (M.-P.R.); ignacio.romagosa@udl.cat (I.R.)

**Keywords:** food barley, phenolic compounds profile, antioxidant capacity, grain development, immature grains, UPLC-MS/MS

## Abstract

Barley is rich in phenolic compounds, providing health benefits and making it a valuable addition to a balanced diet. However, most studies focus on these compounds at barley’s final maturity, neglecting their synthesis during grain development and its impact on barley quality for food applications. This study investigates phenolic profiles during grain development in four hull-less barley genotypes with different grain colors, specifically bred for food applications. The objectives were to determine the phenolic profile and identify the optimal maturity stage for maximum phenolic content and antioxidant capacity. Using UPLC-MS/MS and in vitro antioxidant capacity assays, results show that total phenolic compounds decrease as grain matures due to increased synthesis of reserve components. Flavan-3-ols, phenolic acids, and flavone glycosides peaked at immature stages, while anthocyanins peaked at physiological maturity. The harvest stage had the lowest phenolic content, with a gradient from black to yellow, purple, and blue genotypes. Antioxidant capacity fluctuated during maturation, correlating positively with phenolic compounds, specially bound phenolic acids and anthocyanins. These findings suggest that early harvesting of immature grain can help retain bioactive compounds, promoting the use of immature barley grains in foods. To support this market, incentives should offset costs associated with decreased grain weight.

## 1. Introduction

Barley (*Hordeum vulgare* L.) is a versatile cereal crop primarily used for animal feed and malting, with growing interest as a food source for its health benefits. Barley is rich in bioactive compounds such as dietary fiber, β-glucans, arabinoxylans, and phenolic compounds (PCs) [1]. For human consumption, barleys with high β-glucan content are preferred due to their associated health benefits, such as reducing the risk of cardiovascular disease by lowering blood cholesterol levels [2,3]. Additionally, PCs are valued for their antioxidant properties and potential health benefits including anti-inflammatory, antitumor, and antimicrobial properties [4,5].

PCs are secondary plant metabolites, crucial for defense against stresses and environmental adaptation [6,7], and they provide structural support when cross-linked with cell wall polysaccharides [8]. These compounds may include phenolic acids, anthocyanins, flavonoids, and flavones in barley grain, categorized as free, sugar-conjugated, or insoluble-bound [9]. Insoluble-bound phenolics that predominate significantly contribute to overall antioxidant activity [10]. The color of barley grains is closely linked to their phenolic composition, with flavonoids contributing to yellow, purple, and blue colors, and melanins, derived from polymerized PCs, leading to black grains [11,12]. Purple and blue grains high in anthocyanins, and black grains rich in phytomelanins, have been identified as promising ingredients for the development of cereal-based functional foods due to their natural antioxidant properties [11,13].

During maturation, the physical and chemical compositions of cereal grains undergo substantial modifications affecting nutritional value. Barley grain maturation involves three distinct phases: the initial lag phase with fertilization and rapid cell division, the grain-filling phase with the accumulation of reserve components, and the maturation–drying phase marked by the loss of water content until reaching physiological maturity [14]. 

While cereals are commonly consumed as fully mature grains, immature cereal grains are attracting interest for their nutritional value and digestibility. However, extensive research on the chemical compositions, bioactive compounds, characteristics, and potential applications of immature cereal grains is lacking [15]. Some studies report that immature wheat grains contain more phenols than mature grains [16,17], and whole immature rice grains have higher levels of vitamin B, and tocols [18]. Previous researchers found that immature corn seeds possess high concentrations of reducing sugars, high protein contents with biological activity, and higher antioxidant activity than mature grains [19]. For barley, previous studies showed that total phenolic content gradually decreases throughout grain development, accompanied by a decline in antioxidant capacity [20]. However, comprehensive investigations into the dynamic changes in specific PCs such as phenolic acids, flavan-3-ols, flavones, and anthocyanins, and their corresponding antioxidant activities, during the barley grain development are lacking.

In the present study, four hull-less genotypes differing in grain color and designed for food purposes were selected to investigate the variation in their phenolic profile during grain development. The aim was to identify the grain growth stage with the highest phenolic content and associated antioxidant capacity, to evaluate its potential use as active ingredients with health-promoting properties. To our knowledge, this is the first comparative study of major health-promoting PCs in barley genotypes specifically bred for human food. These findings will contribute to understanding the phenolic dynamics of barley grains, providing valuable insights into the optimal harvesting times to maximize their nutritional value for functional food application.

## 2. Materials and Methods

### 2.1. Experimental Conditions

The field experiment was carried out in collaboration with Semillas Batlle S.A. in Bell-lloc d’Urgell, Lleida, Spain, under irrigation and well-fertilized conditions. The sowing date was 15 December 2022. The genotypes were grown in four main plots sized 4 × 1.5 m^2^ with a seedling rate of 350 seeds/m^2^. Biotic interferences were mitigated by implementing standard practices for weed, insect, and disease control.

### 2.2. Plant Material

Four barley genotypes were used in this study, differing in the number of rows, type of starch, and grain color:

- DHL-190849: Hull-less, two-rowed, normal endosperm, and yellow grain double-haploid advanced breeding line.
- Rajapani^®^: Hull-less, six-rowed, normal endosperm, and blue grain registered Spanish variety.
- DHL-151340: Hull-less, two-rowed, normal endosperm, and purple grain double-haploid advanced breeding line.
- DHL-191250: Hull-less, two-rowed, normal endosperm, and black grain double-haploid advanced breeding line.

One hundred individual spikes of each genotype were marked at the heading to monitor grain growth. At seven-day intervals during grain development (heading until harvest from mid-April to late June), ten spikes were collected from each genotype. Four stages of grain development were selected according to the moisture content of the grains: milky (58–62%), softy (49–51%), physiological maturity (35%), and harvest (12–14%) as described by Ferreira et al. [21]. Spikes were frozen, freeze-dried (Lyobeta 15 TELSTAR Lyophilizer, Terrassa, Spain), and maintained at −20 °C until the analysis. Before the analysis, thousand-grain weight (TGW) was determined with a Marvin system (GTA Sensorik GmbH, Neubrandenburg, Germany) according to the standard MSZ 6367/4-86 (1986) method (Table S1). Then, the grains were milled to a particle size smaller than 0.5 mm (Foss Cyclotec 1093TM mill, Foss Iberia, Barcelona, Spain).

### 2.3. Phenolic Compounds and Anthocyanin Analysis by Ultra-Performance Liquid Chromatography (UPLC-MS/MS)

Free phenols including anthocyanins and bound PCs were extracted from barley according to Martínez et al. [22]. Briefly, 1 mL of 79.5% methanol, 19.5% Milli Q water, and 1% formic acid solution was added to 50 mg of barley flour. The samples were sonicated and centrifuged at 9000× *g* for 10 min. The extraction process was repeated twice more, and the supernatants from the three extractions were pooled and filtered through 0.22 µm polyvinylidene fluoride (PVDF) filter discs before the chromatographic analysis. The residue was hydrolyzed by adding 2 mL of 2 mol/L NaOH to release bound phenols. After leaving the samples at room temperature overnight, they were sonicated for 1 min and centrifuged at 9000× *g* for 10 min. The supernatant was acidified to pH 2 with 37% (*w*/*w*) HCl. Then, 350 µL of the supernatant was mixed with phosphoric acid at 4% and centrifuged again for 10 min, and cleaned up using a micro solid-phase extraction (µSPE) method. The µSPE micro-cartridges were conditioned and equilibrated with methanol and acidified water (pH 2), respectively. After the samples were loaded, the micro-cartridges were cleaned up with water and a Milli-Q water/methanol (95/5, *v*/*v*) solution, and the retained phenolic compounds were eluted with 2 × 50 µL methanol. Finally, the eluate was analyzed by liquid chromatography. Samples were analyzed using AcQuity Ultra-Performance TM liquid chromatography coupled to a tandem mass spectrometer (UPLC-MS/MS) from Waters (Milford, MA, USA) as described by Martínez et al. [22]. Quantification was based on a 0.01–173 mg/L calibration curve of standard compounds with results expressed as μg/g dry samples. Reference standards for the phenolic compound analysis including cyanidin-3-*O*-glucoside (CAS:7084-24-4), luteolin-7-*O*-glucoside (CAS:5373-11-5), apigenin-7-*O*-glucoside (CAS:578-74-5), *p*-coumaric acid (CAS: 614-60-8), and caffeic acid (CAS: 331-39-5) were obtained from Extrasynthese (Genay, France); *p*-hydroxybenzoic acid (CAS: 99-96-7), syringic acid (CAS: 530-57-4), sinapic acid (CAS: 530-59-6), and catechin (CAS: 225937-10-0) were purchased from Sigma-Aldrich (St. Louis, MO, USA); and vanillic acid (CAS: 121-34-6) and ferulic acid (CAS: 1135-24-6) were from Fluka (Buchs, Switzerland). Limits of detection (LODs) ranged from 0.003 to 0.058 mg/L and limits of quantification (LOQs) from 0.010 to 0.173 mg/L. Table S2 shows the selected reaction monitoring (SRM) conditions and the cone voltage and collision energy used for the quantification as well as fragmentation products (MS2) used for the identification of the PCs. This table also indicates the specific phenolic standard compound in which each phenolic has been quantified. PCs were also expressed as μg/grain by multiplying absolute flour measurements by the thousand-grain weight (TGW) and dividing the results by 1000.

### 2.4. In Vitro Antioxidant Capacity

Antioxidant capacity (AC) was measured with the Oxygen Radical Absorbance Capacity (ORAC) according to Huang et al. [23]. Trolox (6-hydroxy-2,5,7,8-tetramethylchroman-2-carboxylic acid) was used as the control. Antioxidant capacity was expressed as μmols Trolox equivalent/g. The antioxidant capacity was determined using a FLUOstar OPTIMA fluorescence reader (BMG Labtech, Ortenberg, Germany) controlled by OPTIMA 2.10R2 software.

### 2.5. Statistical Analysis

All statistical analyses were conducted using JMP^®^ Pro 16 (SAS Institute Inc., Cary, NC, USA). Samples were analyzed in triplicate and the results are presented as the mean value. A general linear model was performed to determine significant differences with genotypes and grain development stages considering fixed and sampling dates’ random factors. Mean comparison tests were assessed by the Tukey–Kramer’s honestly significant-difference tests (HSDs) and *p*-values of less than 0.05 were considered statistically significant. The standard error of the difference (SED) was also included for each variable. 

## 3. Results and Discussion

### 3.1. Deposition Pattern of Total Phenolic Compounds

In this study, total phenolic compound (PC) content in both free and bound fractions was analyzed across distinct stages of grain development in four barley genotypes differing in grain color. Total PCs were quantified as the combined sum of both free and bound extracts and expressed as µg/g of flour (Table 1).

Free, bound, and total PCs exhibited significantly higher levels during the milky and softy immature stages across three of the four studied genotypes. These results align with previous findings in other cereals such as maize, wheat, and rice [17,24,25]. At the harvest stage, significant differences in total PCs were observed among genotypes with black genotype DHL-191250 exhibiting the highest content, followed by yellow, purple, and blue genotypes in decreasing order. These contents surpassed those reported by Ge et al. [26] and Kim et al. [27] for colored barleys, where black genotypes showed the lowest total PC levels compared to blue and purple ones. 

When comparing the total PC content between the immature milky stage and the final harvest stage, a decrease ranging from 35 to 46% across genotypes was observed. Among these, the black genotype DHL-191250 exhibited the smallest. Both total free and bound phenols exhibited different decreasing trends across genotypes during grain maturation, affecting total PCs. In the case of the blue genotype Rajapani^®^, the bound-to-free ratio peaked during the two final maturity stages, exceeding 70%. This fact suggests that during grain development, free PCs decrease drastically compared to bound phenolics, increasing the bound-to-free phenol ratio, as has been reported in wheat [17]. Conversely, the black genotype DHL-191250 showed the opposite trend, with the bound-to-free phenol ratio decreasing as maturation progressed from 49% in the milky stage to 29% at harvest, demonstrating that in this genotype, free phenols did not decline as in the other genotypes. In contrast, the purple genotype DHL-151340 experienced a different trend due to the accumulation of anthocyanins in the central stages of grain development, as will be detailed in subsequent Section 3.2.4. For this genotype, the bound-to-free ratio initially favored bound PCs at the milky stage, starting at 61%. However, once grains reached the softy stage and anthocyanins accumulated, this ratio decreased, reaching a maximum bound-to-free ratio of 52%, then increased again to 76% at harvest.

**Figure 1:**
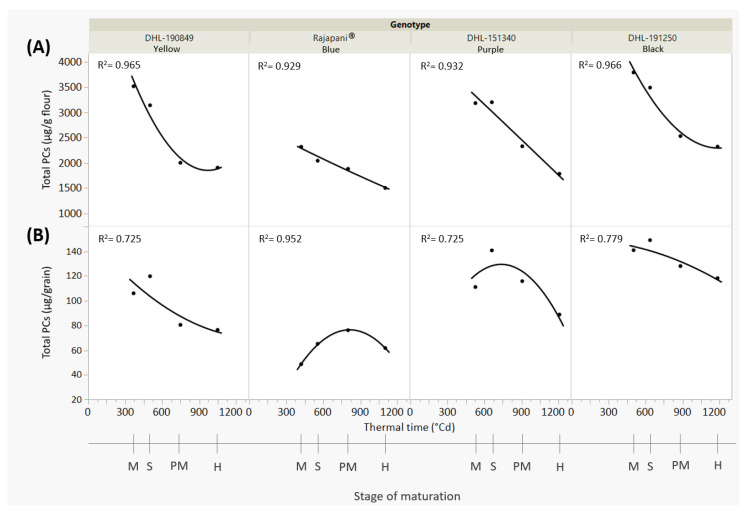
Total phenolic compounds (PCs) of four barley genotypes at different stages of the grain development. (**A**) µg of PCs/g flour. (**B**) µg of PCs/grain. Solid lines represent the best fit (second-degree polynomial curve). M: Milky, S: Softy, PM: Physiological Maturity, H: Harvest. Thermal time (°Cd) was determined using maximum and minimum daily temperatures with a base temperature of 0 °C, using growing degree-days after heading.

To explore the decline in PCs during maturation, it was hypothesized that this could be due to a dilution effect caused by the rise in grain weight during development, as observed in colored wheat genotypes by Ma et al. [28]. Therefore, the total PC levels in flours were assessed relative to their grain content. The results of the total phenolic content per grain showed a deposition pattern different from that observed in flour, with maximum peaks occurring in post-milky stages such as softy or physiological maturity (Figure 1A,B).

These findings suggest a continuous synthesis of some PCs during grain development that can extend until physiological maturity as seen in Rajapani^®^ and the purple genotype DHL-151340. Thus, the significant reduction in PCs from immature to mature stages observed in flour could be attributed to a dilution effect, as starch accumulates in the growing endosperm. Once the grain reaches physiological maturity and grain filling is complete, the decrease in PCs is not significant compared to the harvest stage. However, exceptions are observed in genotypes containing anthocyanins such as Rajapani^®^ and DHL-151340. In these cases, there was a substantial reduction in these compounds from physiological maturation to the harvest stage, contributing to the significant decrease in total PCs. 

Some researchers suggest that the decrease in phenolic content during grain development may result from physiological factors, such as reduced photosynthesis or oxidative metabolism during the grain dehydration process [29]. Several authors have speculated that carbohydrates, such as sucrose, might enhance the activity of phenylalanine ammonia-lyase, an important enzyme during PC biosynthesis, and stimulate phenolic compound production during grain maturation [30,31]. However, as grain filling progresses into the later stages, the availability of carbohydrates could be compromised, particularly during starch synthesis, which could potentially affect phenolic compound formation [28,32]. Wang et al. [33] also reported that the expression of key enzymes such as PAL, C4H, 4CL, CHI, and CHS reaches its maximum levels during early developmental stages, promoting phenolic compound accumulation. In this study, free phenolic compounds decreased drastically towards grain maturation for four genotypes. However, bound phenol content did not follow the same trend for all genotypes. Gruz et al. [34] observed a gradual reduction in free phenolic acid alongside their increase in the insoluble form during the early stages of maturation, suggesting their progressive incorporation into the cell walls. As described later, the main phenols detected in the bound phenolic fraction were phenolic acids, so the increasing in the bound-to-free phenol ratio could be attributed to conversion between fractions (from free to bound) during grain development, and therefore their integration into cell walls. However, as pointed out by other authors [17,24,34], we also observed a subsequent decrease in bound phenolics, which could be caused by the polymerization, oxidation, and conjugation of these components during maturation that, once transformed, would no longer be detectable by spectrophotometry and HPLC–MS [34].

Identifying the optimal developmental stage of cereal grains with the highest contents of PCs is essential from both nutraceutical and pharmaceutical perspectives. PCs, renowned for their antioxidant properties and health benefits, play a vital role in protecting against chronic diseases such as cardiovascular ailments and certain types of cancer [4]. Understanding the peak content of these compounds would allow researchers and the industry to enhance crop management to preserve these compounds in their most beneficial state. This could enhance the nutritional value of food products and facilitate the development of potentially healthy components derived from barley.

### 3.2. Dynamics of Specific Phenolic Compounds across Grain Development Stages

#### 3.2.1. Flavan-3-ols

Flavan-3-ols stand out as a significant category of flavonoids within barley grains [35]. The analysis of the four barley genotypes revealed the presence of 10 different flavan-3-ols, all detected in their free extract. These compounds constituted between 81% and 99% of the total free PCs, as detailed in Table 2.

**Figure 2:**
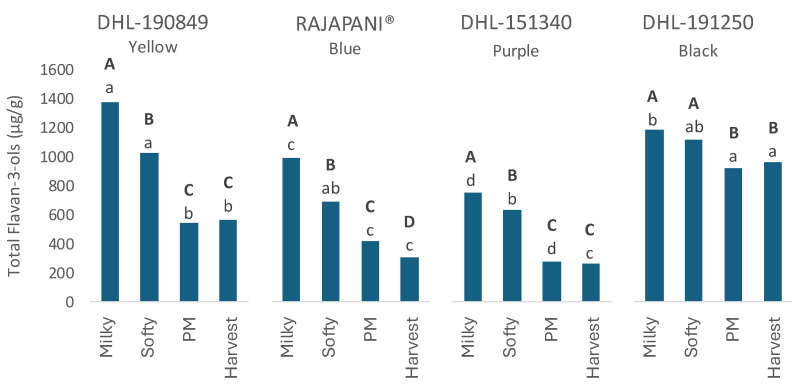
Total flavan-3-ols in flours of four barley genotypes at different stages of the grain development. Statistic evaluation was performed in total flavan-3-ols. Different letters at the same genotype mean significant differences between stages (Tukey–Kramer‘s HSD for α = 0.05). Uppercase letters in bold show the results of tests for differences among stages of maturation within each genotype. Lowercase letters compare mean phenolic compound content across genotypes for individual stages of maturation. PM: Physiological Maturity.

Among the different compounds identified, catechin, procyanidin B3, prodelphinidin B4, and catechin glucoside were the most representative, while other compounds such as procyanidin B3, prodelphinidin B3, procyanidin-diglucoside, procyanidin C2, and some dimers and trimers of these components were detected in minor quantities. A significant decrease in total flavan-3-ols was observed throughout grain development across genotypes as illustrated in Figure 2.

The milky stage of the early development showed the highest content of total flavan-3-ols across genotypes, with yellow genotype DHL-190849 reaching the maximum value followed by the black, blue, and purple genotypes in decreasing order. The greatest reduction in flavan-3-ol content occurred during the immature stages leading up to physiological maturity, reaching a maximum decrease at the harvest stage of 59%, 69%, 65%, and 19% for yellow, blue, purple, and black genotypes, respectively. 

At harvest, the black genotype DHL-191250 exhibited the highest total flavan-3-ol level, reaching 967.6 µg/g of flour, while the purple genotype displayed the lowest with a final level of 264.5 µg/g of flour. These results are consistent with those found by Lin et al. [36], where barley genotypes with black grain showed higher flavonoid content than genotypes with other colors.

Regarding specific flavan-3-ols, all quantified components exhibited a significant decrease with grain development across genotypes, except for procyanidin-diglucoside (Table 2). This compound was not detected in the two immature stages across all genotypes but showed a significant increase with maturation, reaching its peak value at the harvest stage. In contrast, procyanidin C2 remained statistically constant during grain development in both Rajapani^®^ and DHL-191250. Dimers and trimers’ procyanidins were solely quantified in the black genotype, showing no statistical variances between the milky and harvest stages. At harvest, the levels of procyanidin B3 and prodelphinidin B4 in DHL-191250 were notably higher compared to other genotypes, with contents of 324.2 and 375.3 µg/g of flour, respectively. This represents a reduction of 25% and 10%, respectively, from the milky to the harvest stages. In contrast, reductions in other genotypes ranged from 45% to 60% for procyanidin B3, and from 45% to 55% for prodelphinidin B4. This trend may explain why the black genotype DHL-191250 maintains high levels of total flavan-3-ols during maturation compared to other genotypes.

#### 3.2.2. Phenolic Acids and Aldehydes

**Figure 3:**
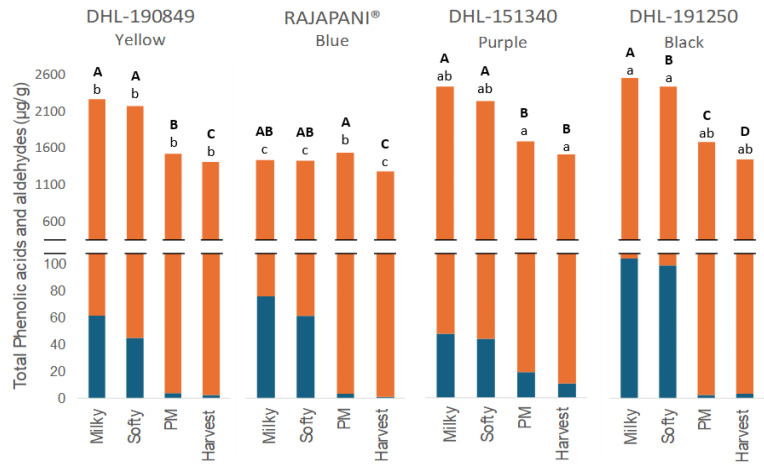
Total phenolic acids and aldehydes in flours of four barley genotypes at different stages of the grain development. Orange bars: Bound extract. Blue bars: Free extract. The statistic evaluation was performed in total phenolic acids. Different letters at the same genotype mean significant differences between stages (Tukey–Kramer‘s HSD for α = 0.05). Uppercase letters in bold show the results of tests for differences among stages of maturation within each genotype. Lowercase letters compare mean phenolic compound content across genotypes for individual stages of maturation. PM: Physiological Maturity.

Phenolic acids, comprising hydroxybenzoic and hydroxycinnamic acids, emerge as the predominant PCs in barley, as noted by Šimić et al. [37]. These acids were detected in both free and bound phenolic fractions across all genotypes, regardless of the maturation stage (Table 2 and Table 3). During grain development, a significant decline in total, free, and bound phenolic acids and aldehydes was evident across genotypes, as illustrated in Figure 3.

At the milky stage, the black genotype DHL-191259 exhibited the highest level of total phenolic acids, followed by purple, yellow, and blue genotypes in descending order. Similar to flavan-3-ols, the most significant decrease in total phenolic acids and aldehydes occurred during the two immature stages leading up to physiological maturity. However, Rajapani^®^ maintained consistent levels of total phenolic acids and aldehydes throughout maturation, experiencing only a slight but significant decrease of 12% from the milky to the harvest stage. At harvest, the purple genotype DHL-151340 showed the highest total phenolic acids and aldehydes, 1470.7 µg/g of flour, while Rajapani^®^ exhibited the lowest, 1201.3 µg/g of flour. These values are consistent with those reported by Holtekjølen et al. [38] for various barley genotypes, which ranged from 604 to 1346 μg/g of flour.

Although there was a significant decrease in total free phenolic acids during physiological maturity and harvest, the predominant presence of total bound phenolic acids ensured that the overall total remained relatively stable, especially during grain development. More than 95% of the total phenolic acids and aldehydes were quantified in the bound extract through the stages of maturity. The ratio of bound-to-free total phenolic acids increased with maturity, rising from 94% in the milky stage to nearly 100% at the harvest stage across all genotypes. This increase can be attributed to the significant decrease in total free PCs during the last two maturity stages across all genotypes, resulting in contents that were almost negligible compared to the total bound phenolic acids. 

Out of the 16 phenolic acids and aldehydes identified, 9 were detected in the free extracts (Table 2). Among these, six were hydroxycinnamic acids, primarily including ferulic acid and its isoform, along with a trimer of it, *p*-coumaric acid, caffeic acid, and sinapoyl-hexose. Additionally, three hydroxybenzoic acids were identified, including *p*-hydroxybenzoic, vanillic, and syringic acids. Total free phenolic acids constituted less than 8% of the total free PCs. Other researchers have also noted that free phenolic acids represent a small portion of the total phenolic acid content in barley [39]. Ferulic acid emerged as the predominant constituent, with contents ranging from 0.9 to 36.4 µg/g of flour in the harvest and milky stages, respectively. As reported by Ou and Kwok [40], ferulic acid is rarely found in the free extract as it is predominantly bound to other compounds in cell walls, such as oligosaccharides and polysaccharides. Sinapoyl-hexose exhibited an even higher content during the milky stage compared to ferulic acid, reaching a maximum content of 47.9 µg/g of flour in the black genotype DHL-191250. Caffeic acid was solely detected in the purple genotype DHL-151340, reaching its peak content at the softy stage and becoming undetectable at the final stage of maturation. 

Concerning hydroxybenzoic acids, syringic acid was only measured in Rajapani^®^ at very low content during the two immature stages of development. Among the phenolic acids identified in the free extract, both hydroxycinnamic and hydroxybenzoic exhibited a significant decreasing trend with grain development, with the majority decreasing in physiological maturation and harvest. It is noteworthy that the presence of *p*-hydroxybenzoic acid in the free fraction was previously linked to colored barley grains as reported by Deng et al. [9]. However, in this study, we detected this compound in its free form across all different genotypes.

Regarding total bound phenolic acids and aldehydes, 15 were detected in the bound extract (Table 3). Across genotypes and maturity stages, total bound PCs were predominantly represented by phenolic acids, consistent with previous findings in barley [22,41,42]. During grain development, ferulic acid emerged as the predominant component, constituting over 60% of the total phenolic acids and aldehydes at the milky stage across genotypes. This percentage increased to 65% at the harvest stage in Rajapani^®^ and the purple genotype DHL-151340. Comparable findings were previously documented in highland barleys at harvest stages [42,43]. Ferulic acid decreased significantly across genotypes from the milky stage to harvest, except in Rajapani^®^. Total bound phenolic acids remained relatively constant during maturation for this genotype, primarily due to the behavior of ferulic acid as the major constituent. Ferulic acid exhibited its highest content peak at physiological maturity in this genotype, reaching 944.4 µg/g of flour. This contrasts with the other genotypes where the milky stage showed the highest content. Iso-ferulic acid, the second major constituent, followed a similar trend. However, distinct patterns were noted for the dimers and trimers of ferulic acids. Di-ferulic, tri-ferulic, and decarboxylated di-ferulic acids exhibited a notable increase from the milky stage to maturation, peaking during the softy and physiological maturity stages before declining towards the final stage of maturity. In Rajapani^®^, decarboxylated di-ferulic acid peaked at the final maturity stage, showing a significant increase from 21 µg/g of flour in the milky stage to 35.2 µg/g of flour in the harvest stage. Similarly, both *p*-coumaric and *m*-coumaric acids displayed a comparable trend, significantly increasing from the milky stage during the intermediate stages of development. Despite the observed trend in these phenolic acids, the dominant presence of ferulic acid and its significant decrease during maturation across genotypes made it challenging to discern the increasing trend of the remaining phenolic acids during the development of total phenolic acids. Among hydroxycinnamic acids, additional compounds with negligible contents were detected, such as sinapic, caffeic, cinnamic, and feruloyl pentose acids. Sinapic acid was exclusively detected in the purple and black genotypes, with contents of 4.2 and 2.7 µg/g of flour, respectively. In contrast, cinnamic acid was found in all genotypes except Rajapani^®^, reaching its highest content in the purple genotype DHL-151240 at 3.5 µg/g of flour. 

Concerning hydroxybenzoic acids, the same three components identified in the free extract were found in the bound extract, including *p*-hydroxybenzoic, vanillic, and syringic acids. Significant differences were observed in hydroxybenzoic acids during maturation, with all constituents experiencing a significant increase across genotypes. Butsat et al. [44] reported a significant increase in bound *p*-hydroxybenzoic, vanillic, and syringic acids during grain development in rice husks. In our study, this increase was particularly pronounced in vanillic acid, which exhibited a content three times higher at harvest compared to the milky stage across genotypes, reaching a maximum value of 9.8 µg/g of flour in the purple genotype DHL-151340. A similar trend was observed in wheat-colored genotypes, where bound vanillic acid reached its peak content at the end of maturation due to the upregulated expression of the TaC3H1 gene associated with its accumulation [28]. Similarly, *p*-hydroxybenzoic acid also showed a significant increase, rising nearly six-fold in the purple genotype DHL-151340 from the milky stage to harvest. Despite the significant increase experienced by hydroxybenzoic acids, their final contribution to total bound phenolic acids was less than 1% across maturity stages. 

Only one aldehyde, syringaldehyde, was identified in the bound extract. This compound showed a notable increase until the harvest stage across genotypes, except in the black genotype DHL-191250, where it peaked at a maximum value of 2.5 µg/g of flour. Consistent with prior research, its overall contribution to total bound phenolic acids was marginal.

#### 3.2.3. Flavone Glycosides

**Figure 4:**
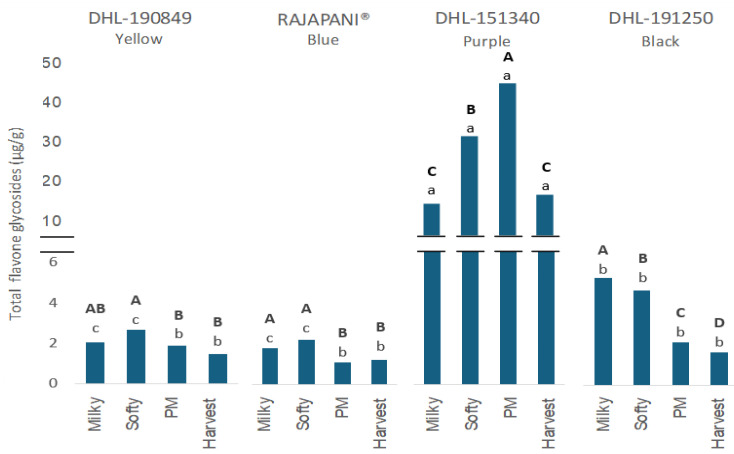
Total flavone glycosides in flour of four barley genotypes at different stages of the grain development. The statistic evaluation was performed in total flavone glycosides. Different letters at the same genotype mean significant differences between stages (Tukey–Kramer‘s HSD for α = 0.05). Uppercase letters in bold show the results of tests for differences among stages of maturation within each genotype. Lowercase letters compare mean phenolic compound content across genotypes for individual stages of maturation. PM: Physiological Maturity.

Figure 4 illustrates the total flavone glycoside values in barley flour across maturation, calculated as the sum of free and bound extracts.

Total flavone glycosides constituted less than 2% of total PCs across genotypes and maturity stages, with the purple barley DHL-151340 showing the highest values, reaching a peak of 45.7 µg/g of flour during physiological maturation. Interestingly, this genotype displayed a distinct trend compared to others, as total flavone glycosides decreased significantly throughout the maturity stages until the harvest stage. This suggests an ongoing synthesis of these compounds in the purple genotype DHL-151340 until physiological maturation.

In the free extract, only two different constituents of total flavone glycosides were detected (Table 2). Among them, luteolin-*O*-glucoside was exclusively detected in the purple genotype DHL-151340, reaching a maximum content of 42.3 µg/g of flour at the physiological maturity stage. At this point, this compound accounts for over 12% of total free PCs, surpassing the relevance of all free phenolic acids to total PCs. This compound has been exclusively detected in the bound extract of colored barleys and minor proportions in the free extract [9]. Isoscoparin-7-*O*-glucoside was the additional constituent identified in this group. Unlike luteolin-*O*-glucoside, this compound was detected across genotypes, peaking at 5.6 µg/g of flour in the black genotype DHL-191250 at the milky stage. In comparison to luteolin-*O*-glucoside, isoscoparin-7-*O*-glucoside demonstrated a significant decrease across genotypes during maturation. Regarding the bound extract, only isoscoparin-7-*O*-glucoside was detected among flavone glycosides (Table 3). 

#### 3.2.4. Anthocyanins

Anthocyanins are water-soluble pigments classified as flavonoids in plants, responsible for the orange, pink, red, violet, and blue colors [45]. Six distinct families of anthocyanins were identified in the blue variety Rajapani^®^ and the purple genotype DHL-151340, as shown in Table 4. 

The purple barley DHL-151340 exhibited significantly higher levels of total anthocyanins in comparison to the blue variety Rajapani^®^, with a peak in physiological maturity of 344.9 µg/g of flour. Both genotypes demonstrated a significant increase in anthocyanin content during maturation, reaching a maximum at physiological maturity. However, this content significantly decreased at the harvest stage, dropping by 81% in Rajapani^®^ and 91% in DHL-151340. Contrary to the proposal by Knievel et al. [46], the possibility of a dilution effect during grain maturation can be discarded, as the reduction in anthocyanin content occurred after the grain filling period concluded. This observation suggests a significant degradation of anthocyanins in the final stages of maturation. Kohyama et al. [47] associated this reduction, occurring just before harvest, in purple hull-less varieties with processes such as the degradation, diacylation, or insolubilization of anthocyanins. Bustos et al. [48] reported a similar pattern in total anthocyanins in purple wheat, while Martínez-Subirà et al. [20] observed the same trend in barley genotypes. In both cases, the peak content of anthocyanins was reached at physiological maturity, followed by a significant decrease until harvest. Additionally, according to Li et al. [49], anthocyanin biosynthesis and accumulation take place during the middle and late filling stages of the grains, followed by degradation until maturity. Among the two anthocyanin-containing genotypes studied, the cyanidin family was predominant. However, in the case of the blue variety Rajapani^®^, the second most represented family was delphinidins, while in the purple genotype DHL-151340, it was pelargonidins. Cyanidin-dimalonylglucoside emerged as the main constituent, followed by cyanidin-malonylglucoside, representing over 70% and 13% of total anthocyanins at the peak content, respectively (Table S3). Similar results were reported by Kohyama et al. [47] in hull-less waxy varieties. Conversely, other authors have reported cyanidin rutinoside as the predominant anthocyanin in other cereals such as wheat, responsible for the purple color [50].

### 3.3. Dynamics of Antioxidant Capacity during Barley Grain Development

**Figure 5:**
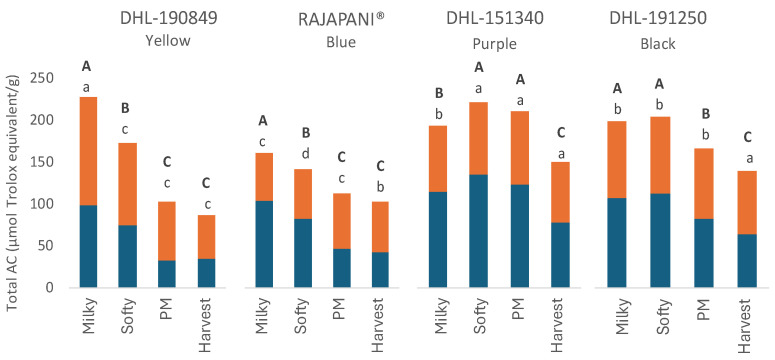
Total antioxidant capacity in barley flours of the four genotypes at different stages of the grain development. Orange bars: Bound extract. Blue bars: Free extract. The statistic evaluation was performed in total AC. Different letters at the same genotype mean significant differences between stages (Tukey–Kramer’s HSD for α = 0.05). Uppercase letters in bold show the results of tests for differences among stages of maturation within each genotype. Lowercase letters compare mean phenolic compound content across genotypes for individual stages of maturation. PM: Physiological Maturity.

The total antioxidant capacity (AC) of barley flour during maturation is depicted in Figure 5. At the milky stage, the yellow genotype DHL-190849 demonstrated the highest total antioxidant capacity (AC) among the examined genotypes, measuring 230.6 µmols Trolox/g. However, it also exhibited the lowest AC across genotypes at the harvest stage, with a value of 86.5 µmols Trolox/g. Consequently, it experienced the most substantial reduction in AC during maturation, decreasing by 62%. A similar trend was observed for Rajapani^®^, which displayed a continuous reduction in AC throughout maturation, reaching the lowest total AC at harvest. In contrast to these two genotypes, purple DHL-151340 and black DHL-191250 genotypes showed an increase in total AC during maturation, reaching peaks of content in the softy stage. The significant rise in AC observed in the purple genotype can be attributed to the increase in anthocyanin content during maturation, as previously reported in barley [51] and maize [52]. In the black genotype, elevated levels of total phenolic acids during immature stages led to a consistent AC during this period. Additionally, the synthesis of phytomelanins in black barley, as suggested by Nowak et al. [11], may enhance AC due to their antioxidant properties. Nevertheless, further research is required to elucidate the role of these components in AC.

In terms of the total AC, the bound and free fractions were not equally represented across the studied genotypes. Despite DHL-190849 and Rajapani^®^ showing a similar decreasing trend in total AC, they exhibited different patterns in the distribution of bound and free fractions during maturation. The yellow genotype DHL-190849 consistently exhibited a higher representation of bound fractions across all maturity stages, with a bound-to-free ratio ranging between 56% and 69% throughout maturation. In contrast, Rajapani^®^ showed a higher proportion of free AC during the immature stages. However, at physiological maturity and harvest, bound AC surpassed free AC in representation. A similar trend to Rajapani^®^ was observed in the bound-to-free AC representation in the black genotype DHL-191250. In the purple genotype DHL-151340, the proportion of free AC predominated over bound AC during all maturity stages, attributed to the predominant content of anthocyanins among all studied PCs during maturation.

At the harvest stage, notable variations in total antioxidant capacity were observed across genotypes. Specifically, the purple DHL-151340 and the black genotype DHL-191250 exhibited the highest antioxidant content, surpassing that of the blue and yellow genotypes. These findings align with those of Dang et al. [53], who reported that purple highland barley had the highest overall antioxidant capacity, followed by black highland barley, with blue and yellow highland barley showing lower levels. The results suggest that the antioxidant capacity of barley is significantly influenced by the grain color, attributed to differences in the composition and content of PCs.

### 3.4. Correlations between Phenolic Compound Families and Antioxidant Capacity across Grain Development

**Figure 6:**
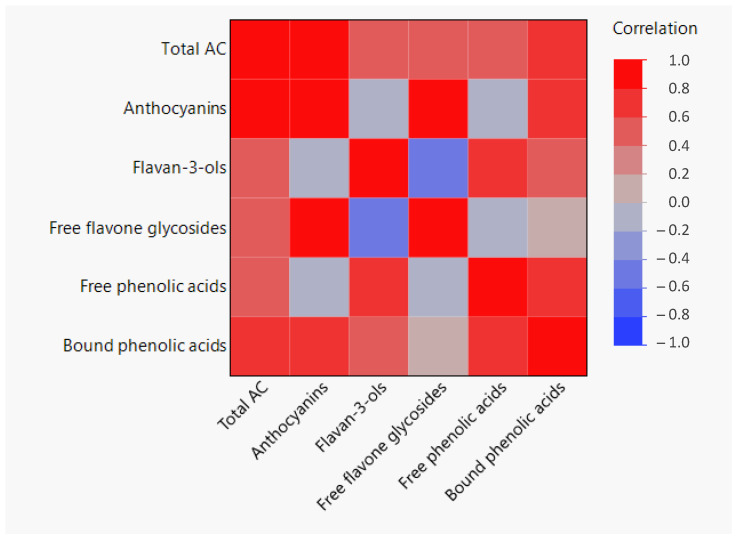
Pairwise correlation heatmap relating the different families of phenols and total antioxidant capacity (AC) analyzed in four barley genotypes during grain development.

Figure 6 shows a heatmap correlation between different PC families and the total AC of the studied genotypes during grain development. Previous studies have highlighted the significant antioxidant activity of PCs in barley [40]. These observations find support in the present study, where AC positively correlated with all detected families of PCs in the barley genotypes. However, the significance level of the positive correlation between AC and phenolic compound families was different. The strongest positive correlation was found between AC and the content of anthocyanins (r = 0.853) and bound phenolic acids (r = 0.775). 

Previous research suggests that the ability of PCs to scavenge free radicals is influenced by their structural composition, such as the presence of methoxy and hydroxyl groups in phenolic acids [54], or the substituents of the heterocyclic and B rings in flavonoids [55]. As was previously commented in Section 3.2.2, bound phenolic acids represent the major constituents among the PCs, contributing significantly to greater AC. Skroza et al. [56] reported that hydroxycinnamic acids exhibit greater antioxidant efficacy compared to hydroxybenzoic acids, with ferulic and sinapic acids displaying better AC than vanillic or syringic acids. Among the bound phenolic acids identified in this study, hydroxycinnamic acids, particularly ferulic acids, and their derivatives were predominant during grain development. Consequently, the robust positive correlation observed between phenolic acids and AC could specifically be attributed to hydroxycinnamic acids. These compounds were predominant in the purple DHL-151340 genotype, followed by the black DHL-191250 genotype, while the yellow DHL-190849 exhibited lower AC despite having similar hydroxycinnamic acid levels to the black genotype (Table 3, and Figure 5). The differences in AC observed between the black DHL-191250 and yellow DHL-190849 genotypes could be attributed to phytomelanins, which are responsible for the black color of the grain. Nowak et al. [11] have reported a strong correlation between antioxidant activity and phytomelanins in black barley genotypes, indicating the strong antioxidant properties of these compounds. Among flavonoids, anthocyanins have been associated with high AC [51], especially when a catechol structure is present in the B ring, as is the case of cyanidin [55]. Our findings support this association, as cyanidins were the predominant anthocyanins identified in barley grains, particularly in the DHL-151340 genotype, which displayed significantly elevated AC. 

Interestingly, flavan-3-ols were negatively correlated with flavone glycosides (−0.454) and a similar trend was observed with anthocyanins that, despite not being significant, showed a low negative value (pairwise correlation equal to −0.101). It was noted that the two genotypes containing anthocyanins, Rajapani^®^ and DHL-151340, exhibited lower levels of flavan-3-ols, particularly in the case of DHL-151340. This genotype also showed the highest content of flavone glycosides. In our previous study, we also observed a pigmented barley genotype containing anthocyanins (Hindukusch), which displayed lower levels of flavan-3-ols and higher levels of flavone glycoside compared to non-pigmented varieties [20]. This pattern is also evident in other crops. Bars-Cortina et al. [57] showed that white-fleshed apples have higher levels of flavan-3-ols than red-fleshed apples. The biosynthesis pathways for flavone glycosides, flavan-3-ols, and anthocyanins share some common steps, particularly involving the precursor compounds in the phenylpropanoid pathway [26]. Although each pathway employs some common substrates and enzymes, there may be competition for these resources within the plant. Deluc et al. [58] suggested that proanthocyanidins (oligomers or polymers of flavan-3-ols) and anthocyanins may accumulate simultaneously if there is enough substrate available, but they may compete when the substrate is lacking. Additionally, regulatory mechanisms within the plant may prioritize the synthesis of certain compounds over others. Zhang et al. [59] indicated that proanthocyanidins and anthocyanins exhibit a competitive relationship in apple peel under light stress, and the inhibition of proanthocyanidin accumulation aids the rapid synthesis of anthocyanins. The negative correlation observed in this study suggests a potential competitive relationship among flavone glycosides, flavan-3-ols, and anthocyanins for shared precursor molecules within the phenylpropanoid pathway, particularly in colored barley. Further research is needed to deepen our understanding of how these flavonoids interact within barley during grain development.

## 4. Conclusions

Four hull-less barley genotypes differing in grain color exhibited distinct phenolic compound profiles among themselves and across different stages of grain development. Notably, immature barley grains exhibited higher contents of PCs in both bound and free extracts compared to mature grains. Moreover, a dilution effect was observed when expressing contents as µg/grain, suggesting ongoing synthesis of PCs during grain development, extending even until physiological maturity, particularly in the Rajapani^®^ variety. 

At the harvest stage, the total PC content followed a gradient from black to yellow, purple, and blue genotypes. However, the maturation process did not uniformly affect all PCs, as evidenced by the divergent trends observed in individual compounds. Particularly, ferulic acid in the bound extract and catechin, procyanidin B3, and prodelphinidin B4 in the free extract emerged as influential contributors to shaping the decreasing behavior of total PCs during grain development. This fact potentially masks the increasing contents of other minor constituents during grain maturation. Anthocyanins reached their maximum peak of content at physiological maturity in blue and purple genotypes, with the latter exhibiting the highest content. Additionally, a significant correlation between PCs and AC was observed, particularly bound phenolic acids and anthocyanins, with the highest values occurring at immature stages, except for the purple genotype, attributed to the high anthocyanin synthesis during the central maturity stages. 

These findings provide valuable insights into the dynamics of phenolic compound accumulation and decline during barley grain development, which is essential for maximizing its nutritional benefits. Additionally, they highlight the potential use of immature barley grains as a functional ingredient in the development of barley-based foods. Notably, the highest levels of PCs in barley grains were reached before the harvest stage, particularly in immature grains. Among the studied genotypes, purple DHL-151340 and black DHL-191250 emerge as the most promising sources of PCs and high antioxidant capacity in their immature stages. Therefore, these genotypes offer significant potential for enhancing the nutritional value and health benefits of barley-based products, making them ideal candidates for further research and development in functional food applications. Consequently, if a market for barley food ingredients develops, early harvesting of non-mature grain should be considered to maximize AC. Possible incentives should be implemented to offset the costs associated with decreased grain weight. Further research is needed to explore these possibilities. 

## Figures and Tables

**Table 1:** Free, bound, and total phenolic content (µg/g flour) in barley flour of four barley genotypes at different stages of grain development.

	DHL-190849 Yellow	Rajapani^®^ Blue	DHL-151340 Purple	DHL-191250 Black
**Free phenolic compounds**				
Milky	1431 A a	1039 A c	922 B c	1284 A b
Softy	1071 B b	757 B c	1036 A b	1223 A a
PM	549 C c	430 C d	689 C b	927 B a
Harvest	568 C b	308 D c	342 D c	972 B a
**Bound phenolic compounds**				
Milky	2106 A b	1282 AB c	2371 A ab	2509 A a
Softy	2079 A b	1289 AB c	2168 A ab	2287 B a
PM	1457 B b	1456 A b	1643 B a	1606 C ab
Harvest	1341 C b	1201 B c	1462 B a	1362 D ab
**Total phenolic compounds**				
Milky	3537 A ab	2321 A c	3193 A b	3793 A a
Softy	3150 B b	2047 B c	3204 A b	3493 B a
PM	2006 C b	1886 B b	2332 B a	2534 C a
Harvest	1909 C b	1509 C d	1788 C c	2325 C a

Mean values followed by different letters are significantly different according to Tukey–Kramer’s HSD for α = 0.05. Uppercase letters show the results of tests for differences among stages of maturation within each genotype. Lowercase letters compare mean phenolic compound content across genotypes for individual stages of maturation. PM: Physiological Maturity.

**Table 2:** Free phenolic compound contents (µg/g) in four barley genotypes at different stages of maturation.

	Flavan-3-Ols									Phenolic Acids									Flavone Glycosides		Total Free Phenols *
	Cat	Cat-g	Pc B3	Pc B2	Pd B4	Pd B3	Pc-d	Pc C2	C-C-GCs	Ferulic	isoF	TriF	*p*-Cm	Caff	Sinap-Hexo	*p*-OHB	Vanill	Syring	Lut-g	Isosc-g	
**DHL-190849 (Yellow)**																					
Milky	469.6 a	87.0 a	346.3 a	14.2 a	476.2 a	21.3 a	nd	10.0 a	nq	15.7 b	7.6 a	1.4 a	1.2 b	nd	31.5 a	1.3 a	3.1 a	nd	nq	2.1 ab	1430.8 a
Softy	216.6 b	75.0 b	303.9 b	6.3 b	403.8 b	20.6 a	nd	7.8 a	nq	19.2 a	6.1 b	1.7 a	1.6 a	nd	13.3 b	1.2 a	3.0 a	nd	nq	2.7 a	1070.5 b
PM	81.8 c	41.0 c	181.2 c	4.0 c	252.1 c	10.6 b	4.7b	4.2 b	nd	2.4 c	nd	nd	nd	nd	nd	nd	1.7 b	nd	nq	1.9 b	549.3 c
Harvest	94.7 c	46.2 c	149.4 d	5.7 bc	234.3 c	11.4 b	7.3 a	3.9 b	nd	1.5 c	nd	nd	nd	nd	nd	nd	1.3 b	nd	nq	1.5 b	567.8 c
*SED*	*11.89*	*2.80*	*9.28*	*0.42*	*12.90*	*0.96*	*0.29*	*0.65*		*0.35*	*0.43*	*0.21*	*0.05*		*2.09*	*0.18*	*0.30*			*0.23*	*28.06*
**Rajapani^®^ (Blue)**																					
Milky	441.5 a	43.3 ab	330.8 a	13.0 a	138.8 a	6.4 ab	nd	9.0 ab	nd	36.4 a	14.7 a	11.6 a	2.1 a	nd	nd	3.5 a	7.3 a	1.2 a	nd	1.7 a	1038.9 a
Softy	308.8 b	32.9 c	211.0 b	8.9 b	112.7 b	7.9 a	nd	7.5 b	nd	30.4 b	16.6 a	7.3 b	2.5 a	nd	nd	2.2 b	4.7 b	1.0 a	nd	2.1 a	754.7 b
PM	41.7 c	45.3 a	199.6 b	7.0 bc	105.9 b	4.3 b	8.3 a	9.8 a	nd	3.2 c	nd	nd	nd	nd	nd	nd	nd	nd	nd	1.0 b	423.3 c
Harvest	33.3 c	42.0 b	138.0 c	5.7 c	71.4 c	1.5 b	7.2 a	7.5 b	nd	0.9 c	nd	nd	nd	nd	nd	nd	nd	nd	nd	1.2 b	306.9 d
*SED*	*12.92*	*0.99*	*10.12*	*0.73*	*5.72*	*0.54*	*0.86*	*0.55*		*0.79*	*1.32*	*0.76*	*0.30*			*0.31*	*0.51*	*0.12*		*0.13*	*29.34*
**DHL-151340 (Purple)**																					
Milky	195.0 a	56.4 a	233.8 a	11.0 a	242.5 a	1.9 a	nd	9.7 a	nd	31.8 a	4.9 a	2.2 a	3.5 a	0.7 b	nd	2.0 b	4.7 ab	nd	10.5 c	2.5 a	815.0 a
Softy	108.5 b	58.5 a	258.9 a	8.7 b	188.7 b	9.7 b	3.9 a	7.4 b	nd	25.9 b	3.8 b	1.2 b	2.7 a	2.1 a	nd	3.1 a	6.7 a	nd	26.7 b	3.1 a	711.6 b
PM	38.4 c	35.8 b	134.2 b	4.1 c	55. 8 d	5.5 c	3.3 a	6.0 bc	nd	10.6 c	1.4 c	nd	1.5 b	0.6 b	nd	1.8 b	4.2 b	nd	42.3 a	2.4 ab	344.2 c
Harvest	24.8 c	43.7 b	129.9 b	4.6 c	81.1 c	1.9 d	6.2 a	4.7 c	nd	6.3 d	nd	nd	nd	nd	nd	0.9 c	3.2 b	nd	13.5 c	1.9 b	311.0 d
*SED*	*4.87*	*3.00*	*7.53*	*0.47*	*3.04*	*0.65*	*0.73*	*0.49*		*0.99*	*0.24*	*0.08*	*0.31*	*0.17*		*0.14*	*0.55*		*1.44*	*0.18*	*7.98*
**DHL-191250 (Black)**																					
Milky	170.5 a	115.6 a	434.3 a	13.7 ab	414.9 a	24.3 a	nd	13.6 b	9.7 a	28.0 a	14.7 a	3.4 a	2.9 a	nd	47.9 a	1.9 a	4.9 a	nd	nd	5.6 a	1283.9 a
Softy	117.5 b	114.2 a	420.4 a	15.4 a	395.0 ab	23.4 ab	nd	16.3 a	7.5 b	30.6 a	15.5 a	1.7 b	2.7 a	nd	41.6 b	0.9 b	5.4 a	nd	nd	4.9 a	1222.6 a
PM	82.2 c	97.8 a	311.2 b	12.0 ab	396.0 ab	19.7 c	17.8 a	12.0 b	10.3 a	1.0 b	1.9 b	nd	nd	nd	nd	nd	nd	nd	nd	2.2 b	927.3 b
Harvest	89.8 c	114.6 a	324.2 b	11.1 b	375.3 b	20.7 bc	19.0 a	13.8 ab	9.1 ab	1.5 b	1.3 b	nd	nd	nd	nd	nd	nd	nd	nd	1.7 b	972.0 b
*SED*	*4.87*	*5.10*	*13.19*	*1.03*	*6.99*	*0.74*	*1.15*	*0.72*	*0.50*	*1.07*	*0.80*	*0.34*	*0.46*		*1.22*	*0.15*	*0.41*			*0.23*	*31.32*

Results are presented as the mean. Means within a column followed by different letters indicate significant differences according to Tukey–Kramer‘s HSD for α = 0.05. SED: standard error of the difference between means. **Flavan-3-ols**: (Cat: Catechin, Cat-g: Catechin-glucoside, Pc B3: Procyanidin, Pc B2: Procyanidin B2, Pd B4: Prodelphinidin B4, Pd B3: Prodelphinidin B3, Pc-d: Procyanidin-diglucoside, Pc C2: Procyanidin C2, C-C-GCs: Sum of C-C-GC/GC-C-C and C-GC-C); **Phenolic acids:** (Ferulic: Ferulic acid, isoF: Iso-ferulic acid, TriF: Tri-ferulic acid, *p*-Cm: *p*-Coumaric acid, Caff: Caffeic acid, Sinap-hexo: Sinapoyl-hexose, *p*-OHB: *p*-Hydroxybenzoic acid, Vanill: Vanillic acid, Syring: Syringic acid); **Flavone glycosides**: (Lut-g: Luteolin-*O*-glucoside, Isosc-g: Isoscoparin-7-glucoside). nd: not detected. nq: not quantified. PM: physiological maturity. * Anthocyanins not included.

**Table 3:** Bound phenolic compound contents (µg/g) in four barley genotypes at different stages of maturation.

	Phenolic Acids															Flavone Glycosides	Total Bound Phenols
	Ferulic	isoF	DiF	TriF	DC DiF	*p*-Cm	*m*-Cm	Sinapic	Caff	Cinna	Ferul-Pen	*p*-OHB	Vanillic	Syring	Syringal	Isosc-g	
**DHL-190849 (Yellow)**																	
Milky	1364.8 a	377.9 a	249.5 ab	21.4 c	28.6 b	9.9 b	0.8 b	nd	0.3 a	nd	nd	1.5 b	1.6 d	0.5 c	0.6 c	nd	2106.3 a
Softy	1418.2 a	423.6 a	255.1 a	37.0 b	36.2 a	9.4 b	0.9 b	nd	0.1 a	nd	0.3 ab	1.6 b	3.0 c	0.7 bc	1.1 b	0.2 a	2078.6 a
PM	866.6 b	258.6 b	211.8 bc	51.8 a	36.5 a	16.5 a	3.8 a	nd	0.2 a	0.4 a	0.4 a	2.5 a	7.0 a	0.9 ab	1.3 a	0.3 a	1457.1 b
Harvest	833.3 b	243.0 b	180.3 c	33.2 b	33.1 ab	7.2 c	0.8 b	nd	0.2 a	0.3 a	0.2 b	1.8 b	5.3 b	1.1 a	1.2 a	0.2 a	1341.1 c
*SED*	*70.59*	*24.57*	*11.16*	*1.75*	*1.94*	*0.64*	*0.06*		*0.04*	*0.04*	*0.04*	*0.10*	*0.31*	*0.10*	*0.05*	*0.03*	*28.01*
**Rajapani^®^ (Blue)**																	
Milky	884.8 ab	229.3 a	122.0 b	10.4 c	21.0 b	12.7 b	1.5 a	nd	0.2 a	nd	nd	1.4 c	1.5 b	0.6 b	1.3 b	0.1 a	1282.4 ab
Softy	823.5 ab	228.3 a	133.7 b	14.5 bc	23.2 b	12.9 b	1.9 a	nd	0.2 a	nd	nd	1.8 b	2.4 b	0.8 ab	0.5 c	0.1 a	1289.3 ab
PM	944.4 a	227.0 a	188.3 a	49.2 a	24.6 b	15.4 a	1.5 a	nd	0.2 a	nd	0.2 a	2.2 a	5.2 a	1.1 a	2.4 a	0.2 a	1456.1 a
Harvest	799.2 b	192.2 a	133.5 b	25.0 b	35.2 a	13.7 ab	1.5 a	nd	0.2 a	nd	0.2 a	2.1 ab	4.9 a	1.2 a	2.3 a	0.1 a	1200.6 b
*SED*	*38.87*	*13.34*	*10.23*	*2.63*	*2.66*	*0.66*	*0.15*		*0.02*		*0.03*	*0.10*	*0.28*	*0.14*	*0.12*	*0.03*	*62.58*
**DHL-151340 (Purple)**																	
Milky	1461.6 a	414.1 a	345.8 b	56.8 b	50.0 b	27.0 bc	2.0 c	3.8 a	0.2 a	3.5 a	nd	1.3 d	2.7 d	0.8 a	1.3 b	0.04 b	2371.1 a
Softy	1240.0 b	328.1 b	398.9 a	82.3 a	63.1 a	32.3 a	3.1 b	4.1 a	0.2 a	3.3 a	1.6 a	2.8 c	5.2 c	0.9 a	1.8 ab	0.07 ab	2167.8 a
PM	1006.0 c	165.4 c	299.4 c	62.0 b	51.6 b	31.7 ab	4.0 a	3.6 a	0.1 a	2.2 b	0.7 b	5.9 b	8.4 b	1.0 a	2.1 a	0.08 a	1642.8 b
Harvest	954.8 c	106.4 d	242.3 d	58.7 b	48.5 b	22.5 c	1.5 c	4.2 a	0.2 a	2.1 b	0.0 b	7.6 a	9.8 a	1.1 a	1.9 a	nd	1461.5 b
*SED*	*39.67*	*8.97*	*7.91*	*4.65*	*2.51*	*1.34*	*0.19*	*0.04*	*0.03*	*0.22*	*0.18*	*0.28*	*0.35*	*0.10*	*0.12*	*0.01*	*61.57*
**DHL-191250 (Black)**																	
Milky	1526.0 a	663.6 a	231.2 b	21.0 b	46.3 b	13.7 b	2.1 b	nd	0.2 a	nd	nd	1.4 a	2.3 d	0.9 a	0.8 c	0.2 a	2509.3 a
Softy	1320.0 b	438.4 b	396.4 a	36.5 a	58.4 a	20.3 a	3.2 a	4.1 a	0.4 a	0.3 a	0.6 a	1.6 a	3.6 c	1.0 a	1.8 b	0.3 a	2286.6 b
PM	960.7 c	347.5 c	220.0 bc	24.8 b	36.3 c	8.5 c	1.2 c	3.8 a	0.2 a	0.2 b	0.3 b	1.4 a	6.4 a	1.0 a	2.5 a	0.3 a	1606.3 c
Harvest	851.4 d	247.2 d	186.2 c	21.2 b	31.2 c	10.5 bc	1.3 c	2.7 b	0.2 a	0.2 ab	nd	1.4 a	5.3 b	1.0 a	1.8 b	nd	1361.6 d
*SED*	*28.59*	*17.86*	*10.75*	*2.37*	*2.52*	*0.87*	*0.17*	*0.27*	*0.07*	*0.01*	*0.04*	*0.12*	*0.21*	*0.13*	*0.07*	*0.05*	*49.85*

Results are presented as the mean. Means within a column followed by different letters indicate significant differences according to Tukey–Kramer‘s HSD for α = 0.05. SED: standard error of the difference between means. **Phenolic acids**: (Ferulic: Ferulic acid, isoF: Iso-ferulic acid, DiF: Di-ferulic acid, TriF: Tri-ferulic acid, DC DiF: Decarboxylated di-ferulic acid, *p*-Cm: *p*-Coumaric acid, *m*-Cm: *m*-Coumaric acid, Sinapic: Sinapic acid, Caff: Caffeic acid, Cinna: Cinnamic acid, Ferul-pen: Feruloyl-pentose, *p*-OHB: *p*-Hydroxybenzoic acid, Vanillic: Vanillic acid, Syring: Syringic acid, Syringal: Syringaldehyde); **Flavone glycosides**: (Isosc-g: Isoscoparin-7-glucoside). nd: not detected. nq: not quantified. PM: physiological maturity.

**Table 4:** Anthocyanin contents (µg/g) in two barley genotypes at different stages of maturation.

Anthocyanin Glycoside and Its Derivates							
	Cyanidins	Pelargonidins	Peonidins	Delphinidins	Petunidins	Malvidins	Total Anthocyanins
**Rajapani^®^ (Blue)**							
Milky	0.29 d	0.01 d	0.01 d	0.06 d	0.01 c	0.001 c	0.37 d
Softy	1.95 b	0.10 b	0.09 b	0.32 b	0.05 a	0.004 b	2.51 b
PM	5.36 a	0.25 a	0.13 a	0.44 a	0.06 a	0.010 a	6.34 a
Harvest	0.85 c	0.04 c	0.02 c	0.23 d	0.02 b	0.001 c	1.16 c
*SED*	*0.054*	*0.002*	*0.030*	*0.005*	*0.001*	*0.000*	*0.054*
**DHL-151340 (Purple)**							
Milky	103.88 c	1.93 b	1.19 c	0.96 c	0.19 b	0.09 c	108.24 c
Softy	301.87 b	14.57 a	2.58 b	3.66 a	0.62 a	0.67 a	323.97 b
PM	323.60 a	13.73 a	3.06 a	3.41 b	0.58 a	0.51 b	344.89 a
Harvest	27.35 d	1.60 b	0.94 d	1.09 c	0.14 c	0.10 c	31.21 d
*SED*	*2.588*	*0.213*	*0.038*	*0.054*	*0.011*	*0.016*	*2.769*

Results are presented as the mean. Means within a column followed by different letters indicate significant differences according to Tukey–Kramer’s HSD for α = 0.05. SED: standard error of the difference between means. PM: physiological maturity.

## Data Availability

The original contributions presented in the study are included in the article/Supplementary Material, further inquiries can be directed to the corresponding authors.

## Supplementary Materials

The following supporting information can be downloaded at: https://www.mdpi.com/article/10.3390/foods13121841/s1, Table S1. Thousand-Grain Weight (TGW) of four barley genotypes at different maturation stages. Table S2. SRM conditions and MS2 fragments were used for the quantification and identification of phenolic compounds in barley samples. Table S3. Anthocyanin contents (µg/g) in four barley genotypes at different stages of maturation.
