# Supplementary material for: Unlocking Phenolic Potential: Determining the Optimal Grain Development Stage in Hull-Less Barley Genotypes with Varying Grain Color
Source: Foods. 2024 Jun 12;13(12):1841. doi: 10.3390/foods13121841 (PMC11202528; doi:10.3390/foods13121841)
Supplement: Supplementary file 1 [file foods-13-01841-s001.zip › foods-3045362-supplementary.pdf]

Supplementary Materials

Unlocking phenolic potential: Determining the optimal grain development stage in hull-less barley genotypes with varying grain color

Iván Friero, Alba Macià, Maria Paz Romero, Ignacio Romagosa, Mariona Martínez-Subirà* and Marian Moralejo*.

Universidad de Lleida – AGROTECNIO-CERCA Center, Av. Alcalde Rovira Roure, 191, 25198 Lleida, Spain

***Corresponding authors:** *E-mail address*: marian.moralejo@udl.cat (Marian Moralejo), mariona.martinez@udl.cat (Mariona Martínez-Subirà). Equally contributed.

Table S1. Thousand Grain Weight (TGW) of four barley genotypes at different maturation stages.

Genotype	Stage	TGW (g)
DHL-190849 (Yellow)	Milky	30.15 ± 0.65
	Softy	38.14 ± 0.16
	PM	40.66 ± 0.30
	Harvest	40.06 ± 0.24
Rajapani® (Blue)	Milky	20.96 ± 0.23
	Softy	31.84 ± 0.46
	PM	40.41 ± 0.98
	Harvest	40.95 ± 2.46
DHL-151340 (Purple)	Milky	34.91 ± 0.92
	Softy	43.98 ± 0.60
	PM	49.73 ± 0.88
	Harvest	48.72 ± 1.76
DHL-191250 (Black)	Milky	37.19 ± 2.38
	Softy	42.71 ± 1.82
	PM	51.60 ± 1.81
	Harvest	50.92 ± 1.42

Results are presented as the mean ± standard deviation. PM: Physiological maturity

Table S2. SRM conditions and MS2 fragments used for the quantification and identification of phenolic compounds in barley samples.

	[M-H]-/+ (*m/z*)	SRM transition[a]	Cone Voltage (V)	Collision Energy (eV)	MS2 fragment spectrum[b]	Quantified as	Identification level[c]
**Flavan-3-ols**							
Catechin	289	245	45	15	245, 205, 151	Catechin	Std
Catechin-glucoside	451	289	45	15	289	Catechin	Tent
Procyanidin B3 (C-C)	577	289	45	20	425,289	Catechin	Tent
Procyanidin B2	577	289	45	20	289	Catechin	Tent
Prodelphinidin B4 (GC-C)	593	289	45	20	289	Catechin	Tent
Prodelphinidin B3 (GC-C)	593	289	45	20	467, 289	Catechin	Tent
Procyanidin-diglucoside	613	451	45	15	451, 289	Catechin	Tent
Procyanidin C2 (C-C-C)	865	289	60	30	577, 289	Catechin	Tent
C-C-GC/GC-C-C	881	577	60	25	577, 289, 245	Catechin	Tent
C-GC-C	881	593	60	25	593	Catechin	Tent
**Phenolic Acids and Aldehydes**							
Ferulic acid (*trans*)	193	134	30	15	178, 134, 149	Ferulic acid (*trans*)	Std
Ferulic acid (*cis*)	193	134	30	15	178, 134, 149	Ferulic acid (*trans*)	Tent
Iso-Ferulic acid	193	134	30	15	178, 134, 149	Ferulic acid (*trans*)	Tent
Di-ferulic acid I	385	341	40	15	341, 326, 282, 297	Ferulic acid (*trans*)	Tent
Di-ferulic acid II	385	341	40	15	341, 326, 297, 282, 267	Ferulic acid (*trans*)	Tent
Di-ferulic acid III	385	341	40	15	341, 326, 282	Ferulic acid (*trans*)	Tent
Di-ferulic acid IV	385	341	40	15	341, 193, 178, 149, 134	Ferulic acid (*trans*)	Tent
Di-ferulic acid (decarboxylated form)	341	282	40	15	326, 282, 267, 297	Ferulic acid (*trans*)	Tent
Tri-ferulic acid I	577	355	50	20	533, 489, 355, 311, 193	Ferulic acid (*trans*)	Tent
Tri-ferulic acid II	577	355	50	20	355, 193	Ferulic acid (*trans*)	Tent
p-Coumaric acid (*trans*)	163	119	35	10	119, 93	*p*-Coumaric acid (*trans*)	Std
Coumaric acid (*m*-CoA or *p*-CoA cis)	163	119	35	10	119, 93	*p*-Coumaric acid (*trans*)	Tent
Sinapic acid	223	164	35	15	208, 164, 149	Sinapic acid	Std
Caffeic acid	179	135	20	12	135	Caffeic acid	Std
Sinapoyl-hexose	385	223	60	25	223, 205	Sinapic acid	Tent
Cinnamic acid	147	103	45	15	103	*p*-Coumaric acid (*trans*)	Tent
Feruloyl-pentose	325	193	40	10	193, 149, 134	Ferulic acid (*trans*)	Tent
p-Hydroxybenzoic acid	137	93	30	15	93	*p*-hydroxybenzoic acid	Std
2.4-Dihydroxybenzoic acid	153	109	45	15	109	*p*-hydroxybenzoic acid	Tent
Vanillic acid	167	123	30	10	152, 123	Vanillic acid	Std
Syringic acid	197	182	30	10	182, 153	Syringic acid	Std
Syringaldehyde	181	166	30	15	166, 151	*p*-Coumaric acid (*trans*)	Tent
**Flavone glycosides**							
Isoscoparin-7-O-glucoside	623	341	60	20	461, 341	Apigenin-7-*O*-glucoside	Tent
Luteolin-7-O-glucoside	447	285	45	25	285	Luteolin-7-*O*-glucoside	Std
**Anthocyanins** *							
Cyanidin-glucoside	449	287	40	20	287	Cyanidin-3-*O*-glucoside	Std
Cyanidin-acetyl-glucoside	491	287	40	30	287	Cyanidin-3-*O*-glucoside	Tent
Cyanidin-malonylglucoside	535	287	40	15	287	Cyanidin-3-*O*-glucoside	Tent
Cyanidin-dimalonylglucoside	621	287	40	20	287	Cyanidin-3-*O*-glucoside	Tent
Pelargonidin-glucoside	433	271	40	20	271	Cyanidin-3-*O*-glucoside	Tent
Pelargonidin-malonylglucoside	519	271	40	25	271	Cyanidin-3-*O*-glucoside	Tent
Pelargonidin-dimalonylglucoside	605	271	40	20	271	Cyanidin-3-*O*-glucoside	Tent
Peonidin-glucoside	463	301	40	20	301	Cyanidin-3-*O*-glucoside	Tent
Peonidin-malonylglucoside	549	301	40	20	301	Cyanidin-3-*O*-glucoside	Tent
Peonidin-dimalonylglucoside	635	301	40	20	301	Cyanidin-3-*O*-glucoside	Tent
Delphinidin-glucoside	465	303	40	20	303	Cyanidin-3-*O*-glucoside	Tent
Delphinidin-malonylglucoside	551	303	40	20	303	Cyanidin-3-*O*-glucoside	Tent
Delphinidin-dimalonylglucoside	637	303	40	20	303	Cyanidin-3-*O*-glucoside	Tent
Delphinidin-rutinisode	611	303	40	20	303	Cyanidin-3-*O*-glucoside	Tent
Petunidin-malonylglucoside	565	317	40	20	317	Cyanidin-3-*O*-glucoside	Tent
Petunidin-dimalonylglucoside	651	317	40	20	317	Cyanidin-3-*O*-glucoside	Tent
Petunidin-rutinisode	625	317	40	20	317	Cyanidin-3-*O*-glucoside	Tent
Petunidin-hexoside-hexoside	691	317	40	20	317	Cyanidin-3-*O*-glucoside	Tent
Malvidin- malonylglucoside	579	331	40	20	331	Cyanidin-3-*O*-glucoside	Tent
Malvidin-dimalonylglucoside	665	331	40	25	331	Cyanidin-3-*O*-glucoside	Tent
Malvidin-hexoside-hexoside	655	331	40	15	331	Cyanidin-3-*O*-glucoside	Tent

[a]SRM transition: selected reaction monitoring used for quantification. [b] MS2 fragment: fragmentation products used for identification. [c] Std: Standard in which the phenolic has been quantified. When the phenolic was not quantified with its own standard, the quantification was tentative (Tent). *Anthocyanins were analysed in positive ion mode.

Table S3. Anthocyanins contents (µg/g) in four barley genotypes at different stages of maturation.

	Rajapani® (Blue)				DHL-151340 (Purple)			
	**Milky**	**Softy**	**PM**	**Harvest**	**Milky**	**Softy**	**PM**	**Harvest**
Cyanidin-glucoside	0.024 ± 0.0042	0.229 ± 0.0142	0.081 ± 0.0025	0.017 ± 0.0009	1.970 ± 0.1483	3.987 ± 0.1187	6.783 ± 0.1197	0.760 ± 0.0154
Cyanidin-acetyl-glucoside	nd	0.008 ± 0.0001	0.005 ± 0.0005	nd	0.188 ± 0.0120	0.478 ± 0.0267	0.539 ± 0.0253	0.072 ± 0.0020
Cyanidin-malonylglucoside	0.107 ± 0.0058	0.771 ± 0.0427	0.806 ± 0.0277	0.131 ± 0.0020	16.179 ± 0.5844	47.423 ± 1.2006	53.368 ± 1.3582	7.021 ± 0.1653
Cyanidin-dimalonylglucoside	0.163 ± 0.0177	0.941 ± 0.0451	4.465 ± 0.1316	0.705 ± 0.0251	85.540 ± 1.8139	249.680 ± 5.0248	262.911 ± 3.2838	19.502 ± 0.2096
Pelargonidin-glucoside	nd	0.004 ± 0.0005	nd	nd	0.033 ± 0.0012	0.147 ± 0.0133	0.202 ± 0.0082	0.045 ± 0.0063
Pelargonidin-malonylglucoside	0.008 ± 0.0004	0.062 ± 0.0014	0.070 ± 0.0027	0.012 ± 0.0003	0.514 ± 0.0195	3.381 ± 0.1965	3.445 ± 0.2110	0.592 ± 0.0258
Pelargonidin-dimalonylglucoside	0.004 ± 0.0003	0.033 ± 0.0010	0.183 ± 0.0044	0.030 ± 0.0016	1.399 ± 0.1360	11.046 ± 0.2629	10.149 ± 0.4383	0.960 ± 0.0596
Peonidin-glucoside	nd	0.013 ± 0.0008	nd	nd	0.222 ± 0.0404	0.493 ± 0.0410	1.045 ± 0.0488	0.437 ± 0.0350
Peonidin-malonylglucoside	0.004 ± 0.0003	0.063 ± 0.0050	0.049 ± 0.0028	0.007 ± 0.0004	0.278 ± 0.0256	0.732 ± 0.0393	0.726 ± 0.0246	0.258 ± 0.0137
Peonidin-dimalonylglucoside	0.002 ± 0.0001	0.016 ± 0.0008	0.080 ± 0.0035	0.012 ± 0.0011	0.693 ± 0.0310	1.356 ± 0.0363	1.299 ± 0.0664	0.241 ± 0.0283
Delphinidin-glucoside	0.007 ± 0.0003	0.027 ± 0.0012	0.033 ± 0.0020	0.019 ± 0.0011	nd	0.378 ± 0.0303	0.442 ± 0.0118	0.099 ± 0.0117
Delphinidin-malonylglucoside	0.051 ± 0.0025	0.216 ± 0.0068	0.293 ± 0.0151	0.161 ± 0.0030	0.566 ± 0.0202	1.916 ± 0.0336	1.666 ± 0.0537	0.885 ± 0.0446
Delphinidin-dimalonylglucoside	nd	0.064 ± 0.0029	0.102 ± 0.0035	0.045 ± 0.0007	0.395 ± 0.0434	1.362 ± 0.0439	1.301 ± 0.0372	0.107 ± 0.0158
Delphinidin-rutinisode	nd	0.009 ± 0.0003	0.008 ± 0.0002	0.004 ± 0.0003	nd	nd	nd	nd
Petunidin-malonylglucoside	0.003 ± 0.0001	0.038 ± 0.0017	0.040 ± 0.0018	0.016 ± 0.0008	nd	0.148 ± 0.0060	0.127 ± 0.0071	0.051 ± 0.0016
Petunidin-dimalonylglucoside	nd	0.012 ± 0.0014	0.013 ± 0.0008	0.004 ± 0.0001	0.133 ± 0.0028	0.399 ± 0.0231	0.373 ± 0.0293	0.052 ± 0.0022
Petunidin-rutinoside	nd	0.004 ± 0.0002	0.003 ± 0.0001	0.002 ± 0.0001	0.029 ± 0.0008	0.024 ± 0.0009	0.035 ± 0.0017	0.020 ± 0.0012
Petunidin-hexoside-hexoside	nd	nd	nd	nd	0.027 ± 0.0026	0.051 ± 0.0006	0.045 ± 0.0030	0.015 ± 0.0006
Malvidin-malonylglucoside	0.001 ± 0.0001	0.004 ± 0.0003	0.007 ± 0.0001	0.002 ± 0.0001	0.075 ± 0.0021	0.412 ± 0.0197	0.364 ± 0.0241	0.059 ± 0.0015
Malvidin-dimalonylglucoside	nd	nd	nd	nd	nd	0.245 ± 0.0191	0.136 ± 0.0123	0.029 ± 0.0032
Malvidin-hexoside-hexoside	nd	nd	nd	nd	0.019± 0.0020	0.019 ± 0.0030	0.012 ± 0.0005	0.011 ± 0.0005

Results are presented as the mean ± standard deviation. PM: Physiological maturity, nd: not detected.
